# The CpG Island in the Murine *Foxl2* Proximal Promoter Is Differentially Methylated in Primary and Immortalized Cells

**DOI:** 10.1371/journal.pone.0076642

**Published:** 2013-10-02

**Authors:** Stella Tran, Ying Wang, Pankaj Lamba, Xiang Zhou, Ulrich Boehm, Daniel J. Bernard

**Affiliations:** 1 Department of Pharmacology and Therapeutics, McGill University, Montréal, Québec, Canada; 2 Department of Medicine, University of California Los Angeles, Los Angeles, California, United States of America; 3 St. Mary Mercy Hospital, Livonia, Michigan, United States of America; 4 Pharmacology and Toxicology, University of Saarland School of Medicine, Homburg, Germany; 5 Department of Obstetrics and Gynecology, McGill University, Montréal, Québec, Canada; 6 Department of Anatomy and Cell Biology, McGill University, Montréal, Québec, Canada; John Hopkins University School of Medicine, United States of America

## Abstract

Forkhead box L2 (*Foxl2*), a member of the forkhead transcription factor family, plays important roles in pituitary follicle-stimulating hormone synthesis and in ovarian maintenance and function. Mutations in the human *FOXL2* gene cause eyelid malformations and premature ovarian failure. *FOXL2/Foxl2* is expressed in pituitary gonadotrope and thyrotrope cells, the perioptic mesenchyme of the developing eyelid, and ovarian granulosa cells. The mechanisms governing this cell-restricted expression have not been described. We mapped the *Foxl2* transcriptional start site in immortalized murine gonadotrope-like cells, LβT2, by 5’ rapid amplification of cDNA ends and then PCR amplified approximately 1 kb of 5’ flanking sequence from murine genomic DNA. When ligated into a reporter plasmid, the proximal promoter conferred luciferase activity in both homologous (LβT2) and, unexpectedly, heterologous (NIH3T3) cells. *In silico* analyses identified a CpG island in the proximal promoter and 5’ untranslated region, suggesting that *Foxl2* transcription might be regulated epigenetically. Indeed, pyrosequencing and quantitative analysis of DNA methylation using real-time PCR revealed *Foxl2* proximal promoter hypomethylation in homologous compared to some, though not all, heterologous cell lines. The promoter was also hypomethylated in purified murine gonadotropes. *In vitro* promoter methylation completely silenced reporter activity in heterologous and homologous cells. Collectively, the data suggest that differential proximal promoter DNA methylation may contribute to cell-specific *Foxl2* expression in some cellular contexts. However, gonadotrope-specific expression of the gene cannot be explained by promoter hypomethylation alone.

## Introduction

Forkhead transcription factors regulate diverse biological processes including embryogenesis, cellular differentiation, cell cycle control, and immune function [1,2]. One family member, forkhead box L2 (*Foxl2*), functions as an essential regulator of ovarian maintenance and function [3-6]. In humans, mutations in the *FOXL2* gene cause blepharophimosis-ptosis-epicanthus inversus syndrome (BPES), a rare autosomal-dominant disorder characterized by eyelid malformations with (type I) or without (type II) premature ovarian failure [3,7-10]. More than one hundred unique *FOXL2* mutations have been described, with the majority clustered in the coding region of the single exon gene [8,11,12]. However, mutations or deletions far upstream or downstream of the coding sequence have also been described and suggest the location of important *cis*-regulatory sequences [e.g., [13-21]].

Similar to the situation in BPES patients, mice harboring loss-of-function mutations in *Foxl2* display cranio-facial and ovarian defects [5,6]. In addition, global or gonadotrope-specific ablation of *Foxl2* causes impaired pituitary follicle-stimulating hormone (FSH) β subunit transcription and FSH synthesis [22,23]. These phenotypes are consistent with *Foxl2*’s restricted pattern of expression in the perioptic mesenchyme of the developing eyelid, ovarian granulosa cells, and pituitary gonadotrope and thyrotrope cells [3,24]. The mechanisms controlling this cell-specific expression have not been described.

To our knowledge, mechanisms of *Foxl2* transcription have only been reported for the caprine (goat) gene. Polled intersex syndrome (PIS) causes the loss of horns (a dominant disorder in both sexes) and sex-reversal (a recessive disorder in females only) in goats [25,26]. PIS is caused by a 11.7 kb deletion on Chr. 1 (syntenic to Chr. 3 in humans) that alters the expression of PIS-regulated transcript 1 (*PSIRT1*; a noncoding RNA), *FOXL2*, and promoter *FOXL2* inverse complementary (*PFOXic*) [27,28]. Interestingly, the effects on transcription are cell-specific, with carriers of the mutation showing increased gene expression in the horn buds, decreased expression in ovary, and no change in expression in the developing eyelids. Remarkably, the PIS mutation affects expression despite the fact that the deletion lies almost 300 kb upstream of the *FOXL2* coding sequence. Though the mechanisms by which this regulatory sequence controls *FOXL2* expression has not been established, the proximal caprine *FOXL2* promoter has been cloned and investigated *in vitro* [28].

A DNA fragment containing 762 bp of 5’ flanking sequence (hereafter proximal promoter) and 293 bp of 5’ untranslated region (UTR) from caprine *FOXL2* confers significant activity to a luciferase reporter (pFOXL2-luc or DK3-luc) when transfected into heterologous COS7 cells. Interestingly, this promoter fragment has activity in both orientations. In the reverse orientation, it appears to drive transcription of *PFOXic*. *PFOXic* is expressed in goats (and other members of the *bovidae* family) but not human or mouse [28]. Wild-type human FOXL2 stimulates DK3-luc activity in homologous KGN cells, suggesting that the gene may be positively autoregulated, at least in ovarian cells [5,29,30]. The reporter is also stimulated by oxidative stress (H_2_O_2_) and heat shock in the same cells [31]. Though these data provide some insight into *FOXL2* transcriptional regulation, they are limited to the caprine promoter and do not directly address mechanisms of cell-specific expression. Here, we characterized the murine *Foxl2* proximal promoter in the homologous gonadotrope-like cell line, LβT2 [32], and in primary gonadotrope cells.

## Materials and Methods

### Reagents

Fetal bovine serum (FBS), normal donkey serum, gentamycin, T4 polynucleotide kinase, Platinum® SYBR® Green qPCR SuperMix-UDG, TRIzol reagent, Plus reagent, Lipofectamine, and Lipofectamine 2000 were from Invitrogen (Burlington, ON). Oligonucleotides were synthesized by IDT (Coralville, IA). Deoxynucleotide triphosphates (dNTPs) were from Wisent Inc. (St-Bruno, QC). Protease inhibitor tablets were from Roche (Indianapolis, IN). 5x Passive Lysis Buffer (PLB), pGL3-Basic, pGEM®-T Easy Vector System kit and GoTaq® Flexi DNA polymerase were from Promega (Madison, WI). Decitabine (5’-aza-2’-deoxycytidine), aprotinin, leupeptin, pepstatin, phenylmethylsulphonylfluoride (PMSF), and the β-actin (A5316) antibody were from Sigma (St. Louis, MO). FOXL2 (IMG3228) antibody was from Imgenex (San Diego, CA). *Foxl2* (siGENOME D-043309-02) and control (siGENOME Non-Targeting siRNA#5; D-001210-05) siRNAs were from Dharmacon (Lafayette, CO). Enzymes *Hha*I, *Hpa*II, McrBC, *M*.*Sss*I and M.*Hha*I were from New England Biolabs Inc. (Ipswich, MA). Gentra Systems DNA Isolation kit was purchased from Bio-Rad (Hercules, CA)

### Cell culture

LβT2 [32], TαT1 [32,33] and GT1-7 [34] cells were gifts from Dr. Pamela Mellon (UCSD, CA). NIH3T3 cells (Todaro and Green, 1963) were from Dr. Patricia Morris (Population Council, NY, NY). AtT20 cells [35] were a gift from Dr. Jacques Drouin (IRCM, Quebec, Canada). TtT/GF cells [36] were obtained from the RIKEN Cell Bank (Japan). C2C12 cells [37] were a gift from Dr. Simon Rousseau (McGill, Quebec, Canada). All cell lines were cultured at 37°C/5% CO_2_ in Dulbecco’s modified Eagle medium (DMEM) with 4.5 g/L glucose (Multicell, Wisent Inc., St-Bruno, QC) and 10% FBS. Additionally, TαT1 cells were cultured on Matrigel (BD Biosciences, Ontario, Canada) diluted 1:20 in serum free media.

### Western blotting

Whole cell protein extracts from cultured cells were prepared in radioimmunoprecipitation assay (RIPA) buffer containing protease inhibitors and western blots performed as previously described [38].

### 5’ rapid amplification of cDNA ends (RACE)

5’ RACE was performed on 1 µg of total RNA from LβT2 cells using the FirstChoice® RNA Ligase Mediated-RACE kit following the manufacturer’s instructions (Ambion/Invitrogen). Following the nested (inner) PCR, A-overhangs were introduced using GoTaq® Flexi DNA polymerase prior to TA cloning (pGEM®-T Easy Vector System kit). Recombinant clones were sequenced by Genewiz (South Plainfield, NJ) using T7 and SP6 primers. Gene-specific primers used in RACE reactions are presented in Table 1.

**Table 1:** Primer sequences (5’–3’).

**Promoter-reporter**	
*Foxl2* -1018 (*Mlu*I site underlined)	GTGCCTTCCACGCGTGACACTTGTGCACGC
*Foxl2* +7 (*Bgl*II site underlined)	CCGAGATCTTTTCCCCCCGGGGAGCGGTCTGC
**5’RLM-RACE**	
*Foxl2* GSP*	CTTTGACTGCGCGTCCGCTCT
*Foxl2* outer GSP	GTCTTCGGGCTCGGGGTAGCT
*Foxl2* inner GSP	CATCATGACAAAAGCCGCTCT
**qAMP**	
*Foxl2* -523/+350.f	CCAGAGGCTTGGATCACCT
*Foxl2* -523/+350.r	ACGGGCGAGTTCATCTCTAA
*Foxl2* -81/+43.f	CCAGAGGCTGACTTCCACTC
*Foxl2* -81/+43.r	GAGGCTGTGCTCTCCTGGT
*Hdc*-595/-361.f	TCCAGCTCTGCTTGCTGTAG
*Hdc*-595/-361.r	TCTGCCCTCACCTCATAGAGA
*Hdc* -79/+303.f	AGGAGCAATCCAAGGGAGAT
*Hdc* -79/+303.r	CTCATTCCCTGCCTCTCATG
**RT-PCR**	
*Foxl2*.f	GCTACCCCGAGCCCGAAGAC
*Foxl2*.r	GTGTTGTCCCGCCTCCCTTG
*Hdc*.f	GATCAGATTTCTACCTGTGG
*Hdc*.r	GTGTACCATCATCCACTTGG

* GSP=gene specific primer

### Promoter-reporter constructs

Once the transcriptional start site (TSS) was identified, we PCR amplified from -1018 to +7 (with the TSS defined as +1) of the *Foxl2* proximal promoter from NIH3T3 cell genomic DNA cells. *Mlu*I and *Xho*I restriction sites were introduced into the 5’ ends of the primers to enable directional ligation into the same sites in pGL3-Basic (Promega). 5’ deletions of the promoter-reporter were generated by introducing *Mlu*I sites at different locations in the parental vector by QuikChange site-directed mutagenesis (Stratagene, La Jolla, CA). The resulting plasmids were digested with *MIu*I, separated from the excised fragments by gel purification, and re-ligated to generate the desired truncated promoter-reporters. To generate *Foxl2* reporter using the CpG-less vector pCpGL-Basic [39] (gift from Dr. Moshe Szyf; McGill University, Montreal, Quebec, Canada), we first linearized the *Foxl2*-pGL3-Basic and pCpGL-Basic vectors by digesting with *Kpn*I and *BamH*I, respectively. Their ends were then blunted with dNTPs and either T4 DNA polymerase or Klenow. Both were then digested with *Hind*III. The *Foxl2* promoter fragment and pCpGL-Basic vector were finally gel purified and ligated together with T4 ligase. Dr. Szyf also provided the pCpGL-CMV/EF1 reporter [39]. Sequences of all plasmids were verified by Genewiz or the McGill University and Genome Quebec Innovation Centre (Montreal, Quebec, Canada). Relevant primer sequences are presented in Table 1.

### Luciferase assays

For luciferase assays, cells were plated in 24-well plates (2.5 x 10^5^ cells per well for LβT2 and 7.5 x 10^4^ cells per well for NIH3T3) 24 h prior to transfection. Cells were transfected with 450 ng of reporter/well using Lipofectamine 2000 following the manufacturer’s instructions. Twenty-four hours after transfection, cells were washed in 1X PBS and then harvested in PLB for luciferase assays as described previously [40]. Luciferase assays were performed on an Orion II microplate luminometer (Berthold detection systems, Oak Ridge, TN) using standard reagents [38].

### CpG methylation analysis by pyrosequencing

Genomic DNA from LβT2, TαT-1, and NIH3T3 cells was extracted using the Gentra Systems DNA Isolation kit and samples sent to EpigenDx (Hopkinton, MA) for CpG methylation analysis. Eight pyrosequencing assays were used (ADS1081-FS1, ADS1081-FS3, ADS1081-FS2 ADS1081-FS1re, ADS1083-FS3, ADS1083-FS2, ADS1082-FS2, ADS1082-FS2, and ADS1084-FS1) to assess 51 CpGs located within the target sequence (murine *Foxl2* -525/+45). Briefly, DNA samples underwent bisulfite conversion followed by PCR amplification to differentiate methylated cytosine ( ^m^C) from unmethyated cytosine (C). Next, pyrosequencing was performed to quantify the ratio of ^m^C:C at individual CpG sites. Results are presented as percent methylation at each CpG of all alleles in the analyzed samples. Cytosines that are not followed by a guanine are used as an internal control for bisulfite treatment and are verified to show 100% conversion to thymine post-treatment.

### Quantitative analysis of DNA methylation by real-time PCR (qAMP)

Genomic DNA was isolated using the Gentra Systems DNA Isolation kit and diluted into 40 ng/µL aliquots. The genomic DNA samples were digested for 5 h at 37°C with the following methylation-sensitive (*Hha*I and *Hpa*II) and methylation-dependent (*McrBC*) enzymes, as described in [41]. The digested DNA samples were then analyzed by quantitative PCR using primers flanking differentially methylated regions of the murine *Foxl2* promoter and containing restriction sites for the above indicated enzymes (see Table 1 for primer sequences). Mean Ct values from the undigested (sham) PCR templates were subtracted from Ct values for the digested templates and the percentage of methylation calculated as described in [41].

### Animals

C57BL/6 mice were purchased from Charles River (Montreal, Canada), housed on a 12L:12D light cycle and were given *ad libitum* access to food and water. Adult male and female mice were euthanized with carbon dioxide, organs (heart, liver and testis from one male; ovary from a female) were harvested and washed in cold PBS. Genomic DNA was extracted using the Gentra Systems DNA Isolation kit according to the manufacturer’s instructions. In a separate experiment, gonadotropes were genetically labeled with yellow fluorescent protein (YFP) as described in [42]. In this model, a gonadotrope-specific Cre driver is used to excise a transcriptional stop cassette (lox-STOP-lox), allowing for YFP expression from the *Rosa26* locus. Gonadotropes (YFP+) and non-gonadotropes (YFP-) were then isolated from dissociated pituitaries by fluorescence activated cell sorting as described in [22,43]. All animal procedures were approved by the Animal Care and Use Committee of McGill University.

### In vitro methylation

The -432/+7 *Foxl2* promoter or CpG-less CMV/EF1 promoter in pCpGL-Basic was incubated at 37°C for 5 h with the methytransferase enzymes *M*.*Sss*I or M.*Hha*I in the presence of methyl group donor S-adenosylmethionine (SAM). Mock-methylated plasmids were incubated without enzyme but in the presence of SAM. Samples were purified by ethanol precipitation before transfection. Diagnostic digests were performed on the mock-methylated and methylated plasmids with HpaII, a methylation-sensitive enzyme, and its methylation-insensitive isoschizomer MspI to confirm the efficacy of the *in vitro* methylation.

### Decitabine (5-Aza-2’-deoxycytidine) treatment

Prior to Day 1 of treatment, NIH3T3 cells were seeded sparsely (~5 x 10^5^ cells) into 10-cm dishes and allowed to attach overnight. On Day 1, cells were treated with serum-free DMEM containing 10 µM of decitabine (in DMSO) or vehicle. Because decitabine is unstable in solution, cultures were re-fed with fresh decitabine and 10% FBS in DMEM at days 3 and 6. Cells proliferated normally with minimal cell death. On Day 8, RNA was collected using TRIZOL and DNA was harvested using the Bio-Rad DNA Isolation kit. RNA and DNA were extracted from duplicate plates treated at the same time. Decitabine treatment had no apparent effects on cell proliferation or viability.

### RT-PCR

RNA was extracted from the indicated cells using TRIzol following manufacturer’s instructions. Reverse transcription was performed on 2 µg of DNAse-treated RNA as previously described [44]. Primer sequences are shown in Table 1.

### Statistics

Statistical analyses were performed as indicated in the figure legends using GraphPad Prism 5 software (GraphPad, La Jolla, California). Data were log transformed prior to analysis when variances were unequal. Significance was assessed relative to *p* <0.05.

## Results

### The murine *Foxl2* proximal promoter confers reporter activity in both homologous and heterologous cell lines

**Figure 1:**
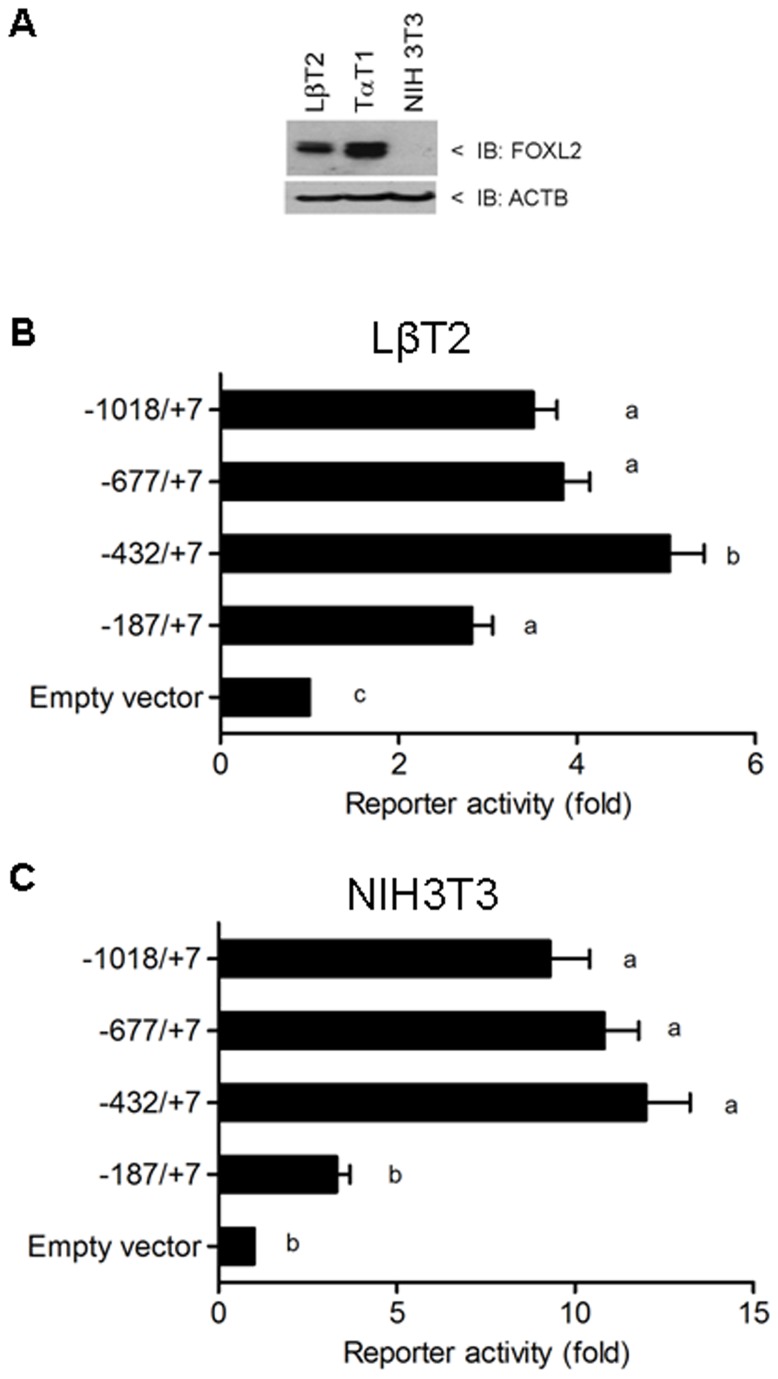
The murine *Foxl2* 5’ flanking sequence possesses promoter activity in both homologous and heterologous cells. A) FOXL2 protein expression in immortalized murine cell lines: gonadotrope-like (LβT2), thyrotrope-like (TαT-1) and fibroblast-like (NIH3T3). ACTB, β-actin B) LβT2 cells were transfected with the indicated murine *Foxl2* promoter-reporters or the empty vector, pGL3-Basic. Transcriptional activity was measured using luciferase assays. The data reflect the means of 16 independent experiments (+SEM) and are presented with empty vector activity set to 1. C) NIH3T3 cells were transfected and analyzed as in panel B. Here the data are from 11 independent experiments. In panels B and C, data were analyzed by one-way ANOVA followed by Tukey *post-hoc* tests. Bars with different letters were statistically different, whereas those sharing letters did not differ.

During murine embryogenesis, FOXL2 expression is first detected at embryonic day 11.5 in the developing pituitary, co-localizing with the glycoprotein hormone subunit α (*Cga*), a marker of the gonadotrope and thyrotrope lineages. In adulthood, *Foxl2* is expressed in the gonadotrope and thyrotrope cells of the anterior lobe [24]. Consistent with this *in vivo* pattern of expression, we detected endogenous FOXL2 protein expression in immortalized murine gonadotrope-like (LβT2) and thyrotrope-like (TαT1) cell lines (Figure 1A). In contrast, the murine fibroblast cell line, NIH3T3, does not express FOXL2.

**Figure 2:**
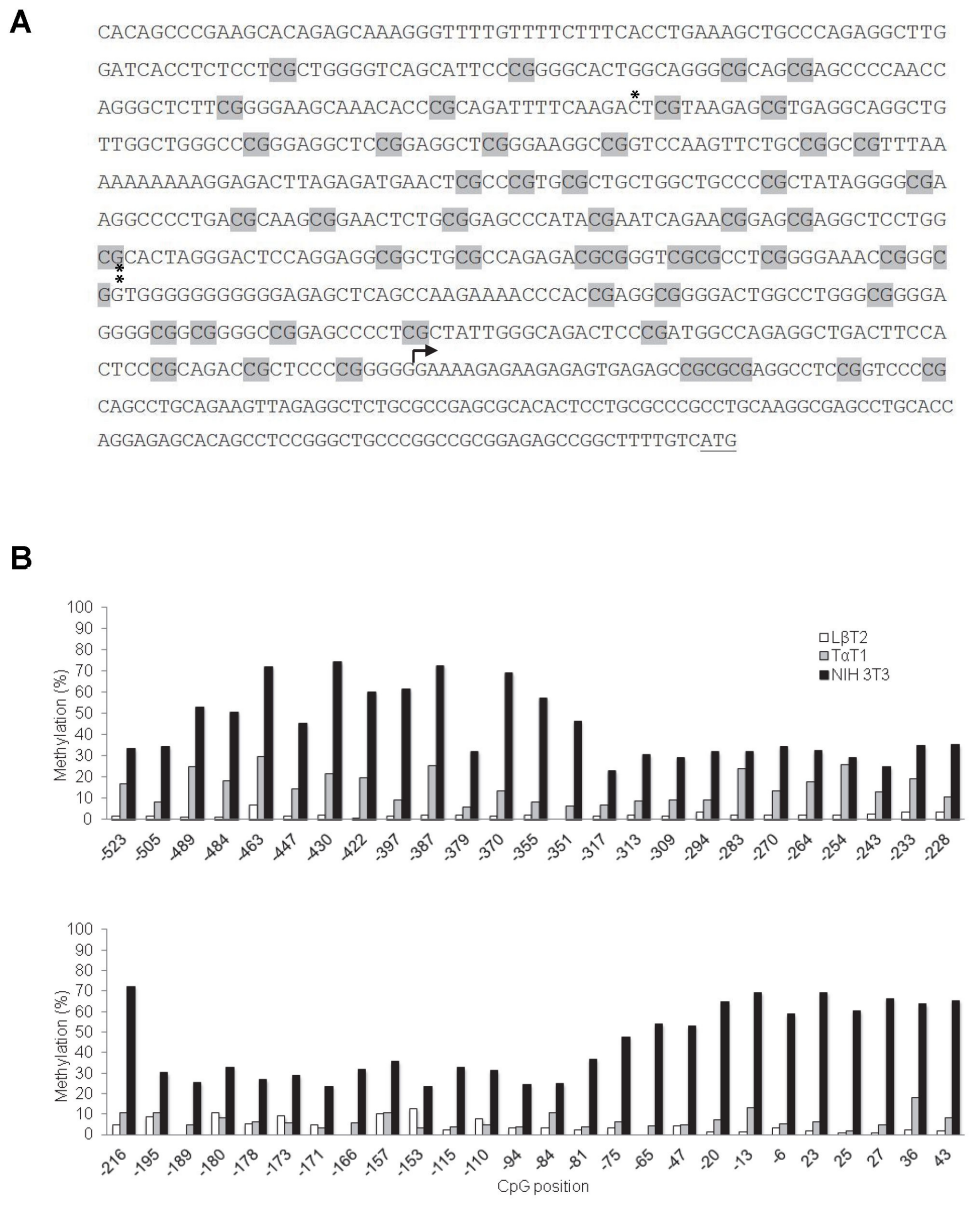
The murine *Foxl2* promoter is differentially methylated in homologous and heterologous cells. A) DNA sequence (-600 to +165) of the murine *Foxl2* promoter. Of 62 CpG dinucleotides pictured, 51 were analyzed by pyrosequencing (shaded in grey). The transcriptional start site (+1) mapped here by 5’ RACE is indicated with an arrow. The ATG translation initiation codon is underlined. The first nucleotides of the -432 and -187 reporters are marked above the sequence with * and **, respectively. B) Percent methylation of the CpGs at the indicated positions in genomic DNA from LβT2 (white bars), TαT1 (grey bars), and NIH3T3 (black bars) cell lines as assessed by pyrosequencing.

Given our and others’ previous research on FOXL2 function in gonadotrope cells [22,23,40,45-50], we investigated mechanisms of gonadotrope-specific *Foxl2* expression. To this end, we first mapped the *Foxl2* transcription start site (TSS) in LβT2 cells using 5’ RACE. The identified TSS was positioned 165 base pairs (bp) upstream of the start of translation (data not shown; but see Figure 2A). We then PCR amplified from -1018 to +7 (where +1 is the first base of TSS) of the proximal promoter from murine genomic DNA and ligated it upstream of a luciferase reporter in pGL3-Basic. When transfected into LβT2 cells, the -1018/+7 fragment conferred reporter activity relative to the empty parental vector (Figure 1B). As described above, the *PFOXic* transcript identified in goat appears to be absent in mouse [28]; however, a promoter fragment (-677/+7, see more below) ligated in the antisense orientation similarly conferred reporter activity in these cells (Figure S1A). These data indicated that the murine *Foxl2* 5’ flanking sequence possesses promoter/enhancer-like activity in homologous cells. As the caprine promoter was reportedly induced by FOXL2 (autoregulation) and oxidative stress [29,31], we examined the effects of FOXL2 overexpression and knockdown as well as H_2_O_2_ exposure on murine *Foxl2* reporter activity. None of these treatments altered transcriptional activity in our experiments (data not shown).

To identify the minimal *Foxl2* promoter, we generated and transiently transfected 5’ deletion constructs into LβT2 cells. All truncated reporters, with the exception of -187/+7, had comparable or greater activity to the ~1 kb reporter (Figure 1B). The -187/+7 construct conferred greater activity than the empty vector, but less than the next shortest construct tested, -432/+7. The latter was therefore considered the minimal promoter. As unique proteins or sets of proteins often dictate cell-specific gene expression, we next assessed whether the murine *Foxl2*-luc reporters were active in heterologous cells. Remarkably, the various *Foxl2* promoters exhibited similar activities when transfected into NIH3T3 compared to LβT2 cells (Figure 1C; see also Figure S1B). These results suggest that the transcription factors required for induction of the murine *Foxl2* proximal promoter are not unique to homologous cells. This is also the case with the caprine promoter, which is similarly active in heterologous cells [28].

### The *Foxl2* proximal promoter is hypermethylated in NIH3T3 cells

**Figure 3:**
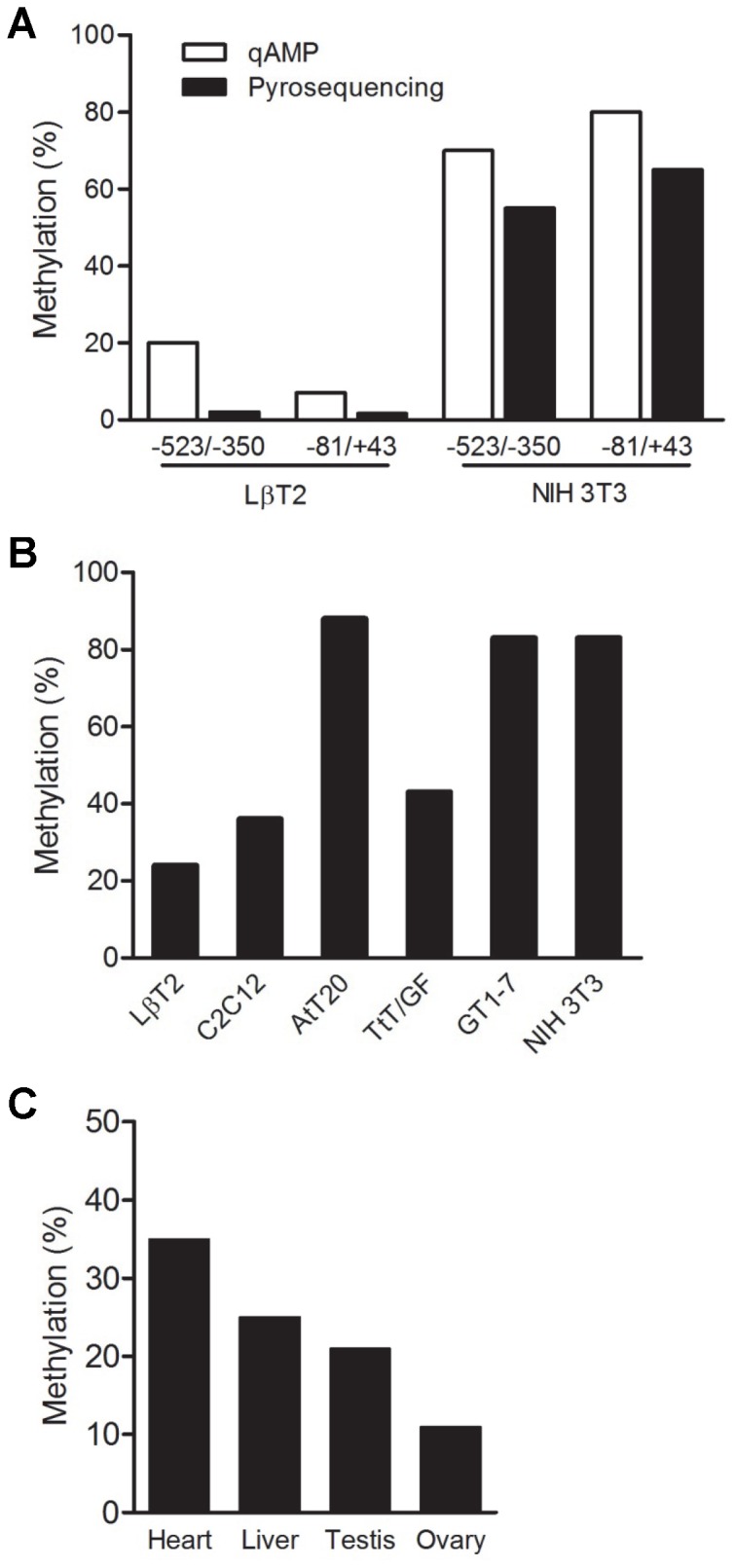
The *Foxl2* promoter is hypomethylated in homologous cells and tissues. A) Analysis of percent methylation of two regions of the *Foxl2* promoter in genomic DNA from LβT2 and NIH3T3 cells as assessed by qAMP (white bars) and pyrosequencing (black bars). Data are from a representative of a 2 replicate experiments, which yielded comparable results. B) Percent methylation of -81/+43 of the *Foxl2* promoter in genomic DNA from the indicated murine cell lines. Data are from a single experiment. C) Percent methylation of -81/+43 of the *Foxl2* promoter in genomic DNA from the indicated murine tissues Data are from a representative of a 2 replicate experiments, which yielded comparable results.

As the *Foxl2* promoter-reporter but not endogenous *Foxl2* was active in NIH3T3 cells, we asked whether the gene might be epigenetically regulated. Analysis of the proximal promoter sequence revealed a high density of CpG dinucleotides (Figure 2A). Indeed, by virtue of being greater than 200 bp in length, possessing ~68% GC content, and having an observed to expected CpG ratio of 0.8, the region spanning from -523 through +44 qualifies as a CpG island [51]. We therefore used pyrosequencing to measure the relative methylation of the individual CpGs within this region in genomic DNA from LβT2, TαT1, and NIH3T3 cells. In general, the 51 CpGs analyzed between -523 and +44 were hypermethylated in NIH3T3 cells relative to the two homologous cell lines (Figure 2B). We confirmed these results in LβT2 and NIH3T3 cells by quantitative analysis of DNA methylation using real-time PCR (qAMP) (Figure 3A). TαT1 cells were not analyzed by qAMP because of difficulties maintaining this cell line in culture.

### DNA methylation silences proximal *Foxl2* promoter activity

**Figure 4:**
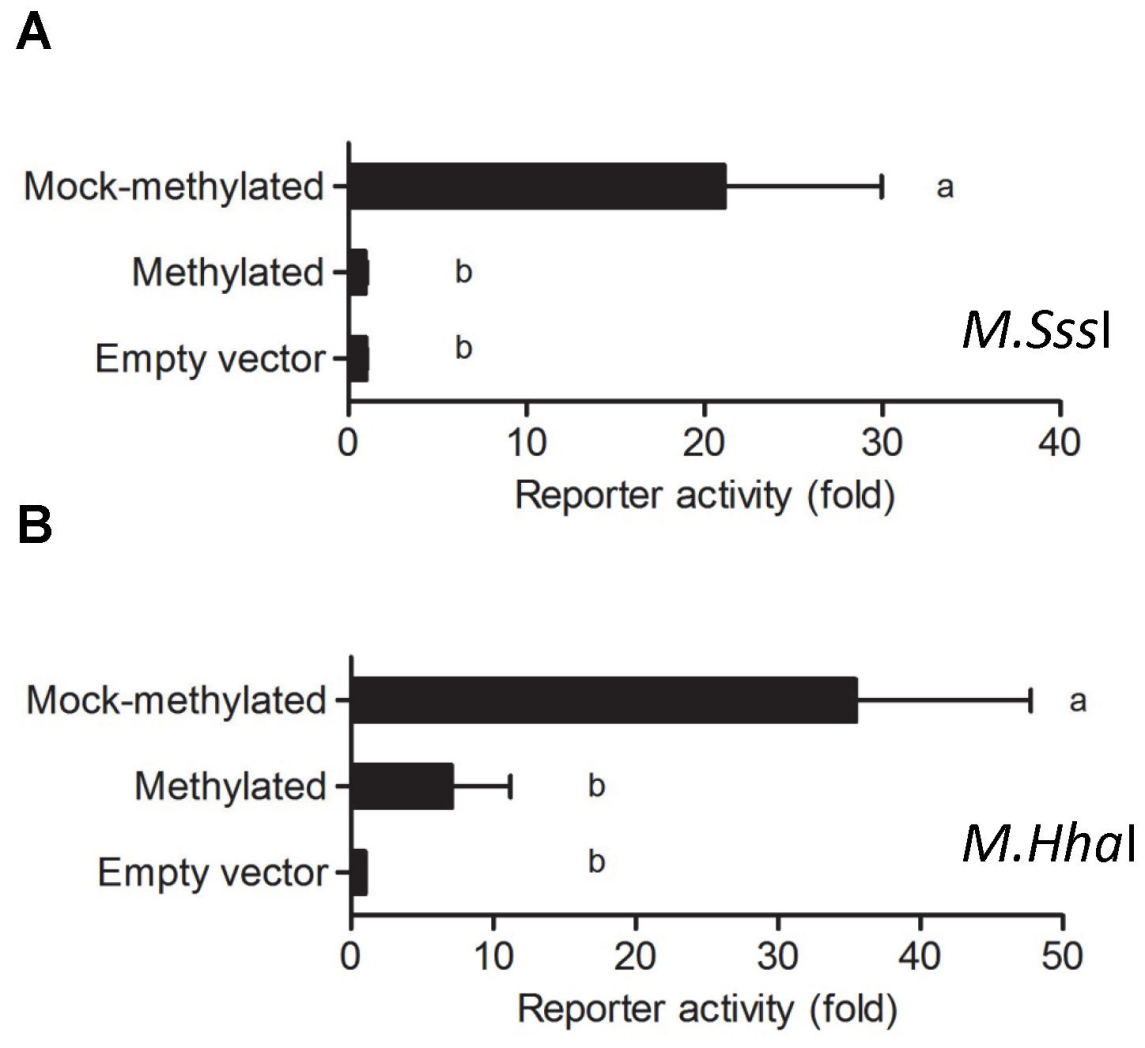
*In vitro* methylation silences *Foxl2* promoter reporter activity in NIH3T3 cells. The -432/+7 murine *Foxl2*-luciferase reporter in pCpGL-Basic was treated *in* *vitro* with A) *M*.*Sss*I or B) M.*Hha*I. Mock methylated plasmid was exposed to the identical treatment but without enzyme. Plasmids were transfected in triplicate into NIH3T3 cells. For A (n=6) and B (n=4), the data reflect the means of independent experiments (+SEM) and are presented with empty vector activity set to 1. Data were analyzed by one-way ANOVA followed by Tukey *post-hoc* tests. Bars with different letters were statistically different, whereas those sharing letters did not differ.

To investigate whether the *Foxl2* proximal promoter is silenced via DNA methylation, we transferred the minimal promoter fragment (-432/+7) into a luciferase reporter devoid of CpG dinucleotides [pCpGL-Basic, as described in [39]]. We then incubated the construct with *Sss*I (*M*.*Sss*I) to methylate the cytosines in the 40 CpGs within the -432/+7 interval. Promoter methylation was confirmed by methylation-sensitive restriction digest (data not shown). Compared to the mock-methylated control, the *in vitro* methylated -432/+7 *Foxl2*-Luc reporter was completely silenced in both LβT2 (Figure S2A) and NIH3T3 cells (Figure 4A). Treatment of pCpGL-CMV/EF1, which lacks CpG dinucleotides, did not suppress promoter activity in either cell line (Figure S2), demonstrating the specificity of the methylation reaction. We extended the analysis in NIH3T3 cells with a second methyltransferase, *Hha*I (M.*Hha*I), which methylates the first cytosine in the sequence 5’-GCGC-3’. There are four such motifs within the -432/+7 interval. Remarkably, M.HhaI reduced promoter activity by 70% relative to the mock methylated control (Figure 4B). Thus, even minimal methylation can strongly suppress *Foxl2* transcription.

To test the hypothesis that *Foxl2* is epigenetically silenced in NIH3T3 cells, we treated cells with decitabine (5’-Aza-2’-deoxycytidine), a DNA methyltransferase 1 (DNMT1) inhibitor that passively demethylates CpG dinucleotides in replicating cells [52]. We treated cells for 8 days to allow multiple rounds of cell division, and then collected RNA and DNA for analysis of gene expression and promoter methylation, respectively. Decitabine reportedly demethylates the L-histidine decarboxylase (*Hdc*) promoter, inducing the gene’s expression in NIH3T3 cells [53]. We replicated those results here (Figure S3A, lane 2 of the middle panel; and Figure S3B), confirming the activity of the inhibitor in our experiments. In contrast, decitabine failed to induce *Foxl2* promoter demethylation (Figure S3C) or mRNA expression in NIH3T3 cells (Figure S3A, lane 2 of the upper panel). LβT2 cell RNA was included in the expression analyses as a positive control for the *Foxl2* primer set (Figure S3A, lane 3 of the upper panel). Some epigenetically silenced genes are only activated upon co-administration of DNMT and histone deacetylase (HDAC) inhibitors [54-57]. We therefore treated NIH3T3 cells with decitabine and trichostatin A alone and in combination. Again, this failed to induce *Foxl2* expression (data not shown). Therefore, it remains unresolved whether DNA demethylation is sufficient to activate the *Foxl2* promoter in NIH3T3 cells and, indeed, whether DNMT1 is the relevant *Foxl2* methyltransferase in these cells [e.g., [58-63]].

### Differential proximal promoter methylation does not explain patterns of *Foxl2* expression in all cells

**Figure 5:**
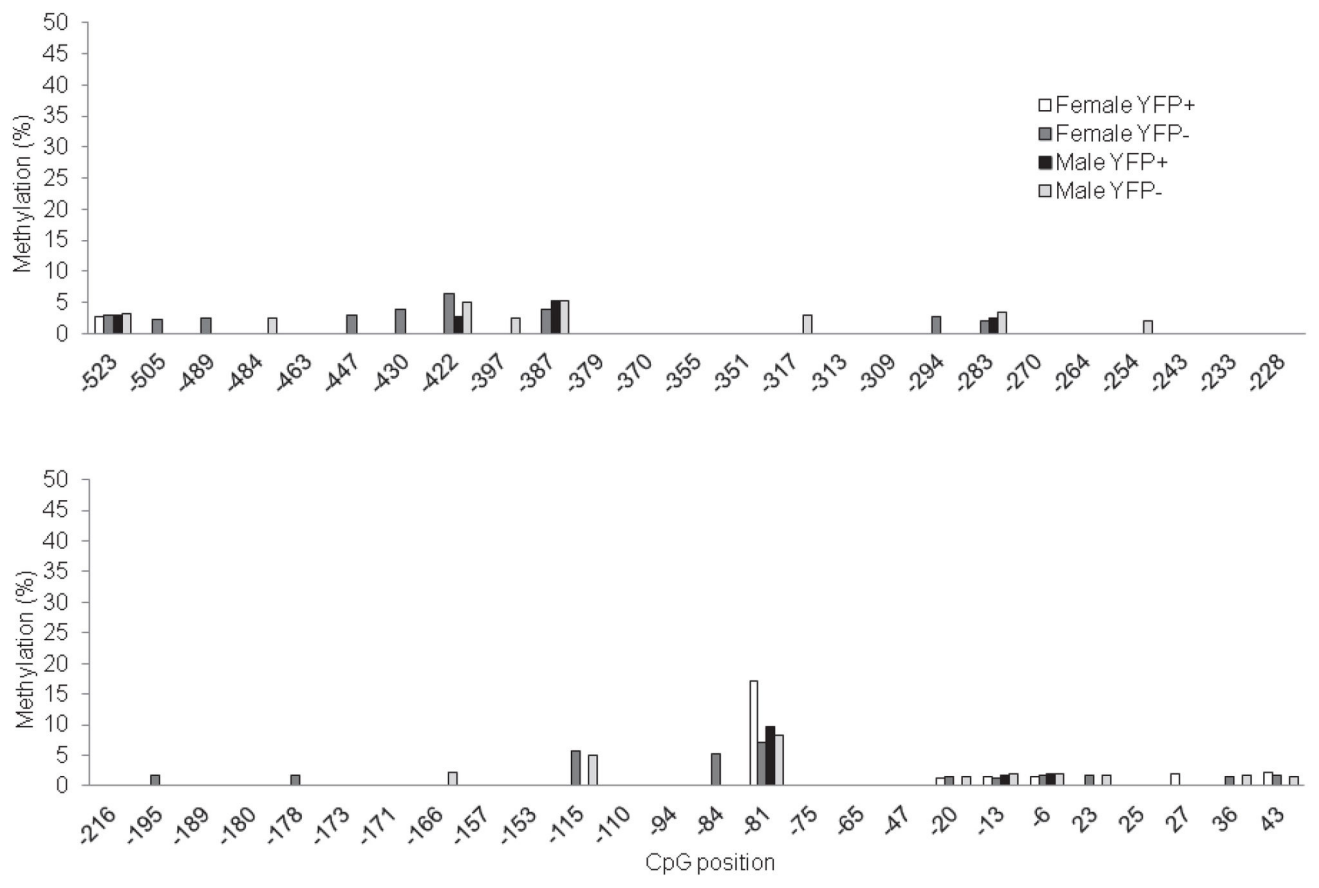
The *Foxl2* promoter is hypomethylated in purified murine pituitary cells. DNA was extracted from genetically labeled gonadotropes (YFP+) or non-gonadotropes (YFP-) of adult male and female mice. The data show percent methylation of the indicated CpGs in the *Foxl2* promoter as assessed by pyrosequencing.

We next assessed whether proximal promoter hypermethylation might represent a general mechanism of *Foxl2* gene silencing in heterologous cells. For these analyses, we investigated a mix of pituitary and non-pituitary-derived cell lines: AtT20 (corticotrope-like), C2C12 (myoblast-like), GT1-7 (GnRH neuron-like), and TtT/GF (folliculostellate-like). We confirmed that these cell lines do not express *Foxl2* mRNA or FOXL2 protein (Figure S4). Similar to NIH3T3 cells, AtT20 and GT1-7 cells showed promoter hypermethylation (Figure 3B). In contrast, C2C12 and TtT/GF were hypomethylated relative to NIH3T3 cells, though still showed apparently greater methylation compared to LβT2 cells. For the qAMP analyses in these additional cell lines, we focused on the proximal promoter and 5’ UTR (-81/+43), assuming that the data for this region would also reflect the pattern of methylation more distally (-523/-350), as we saw in LβT2 and NIH3T3 cells (Figure 3A). To extend these observations to more physiological conditions, we first analyzed the *Foxl2* promoter in homologous (ovary) and heterologous (heart, liver, testis) murine tissues. The promoter appeared to be hypomethylated in ovary relative to the other tissues (Figure 3C). Finally, we examined *Foxl2* promoter methylation by pyrosequencing in purified gonadotropes relative to other pituitary cell types. Similar to what we observed in LβT2 cells, the promoter was hypomethylated in gonadotropes of both adult male and female mice (Figure 5). Interestingly, the promoter was also hypomethylated in non-gonadotrope cells, which included thyrotropes.

## Discussion

Methylation of cytosines within CpG dinucleotides is a well-described mechanism of gene silencing in mammals [56,64-66]. The identification of a CpG island within the murine *Foxl2* proximal promoter and 5’ UTR, combined with pronounced *Foxl2* promoter-reporter activity in heterologous NIH3T3 cells, prompted us to investigate whether tissue/cell-specific *Foxl2* expression might be regulated by DNA methylation. Using two methods, pyrosequencing and qAMP, we observed *Foxl2* promoter hypomethylation in a gonadotrope-like and thyrotrope-like cell lines and in purified gonadotropes, where the gene is normally expressed [e.g., [5,24]]. In contrast, the *Foxl2* promoter was hypermethylated in heterologous cell lines (e.g., NIH3T3, AtT20, and GT1-7) and non-expressing tissues such as the heart and testis. We further showed that methylation of as few as four CpGs within the proximal promoter significantly inhibits *Foxl2* promoter-reporter activity. Therefore, differential proximal promoter methylation may contribute to cell-restricted expression of the *Foxl2* gene, at least in some cell types. Tissue- and cell-restricted expression via DNA methylation has also been reported for another important gonadotrope- and granulosa-cell specific transcription factor, *Nr5a1* (SF1) [67,68].

By 5’ RACE, we mapped the *Foxl2* transcription start site in LβT2 cells to 165 bp upstream of the translation initiation codon. This is longer than the 5’ UTR (54 bp) currently indicated for murine *Foxl2* in GenBank (acc. # NM_012020). It should be noted, however, that the means by which the UTR was mapped and the source material of the cDNA are not clearly indicated for the latter. Two murine ovarian expressed sequence tags (ESTs) corresponding to *Foxl2* have 5’ UTRs that map 175 (acc. # BB666619) or 178 bp (acc. # BB666604) upstream of the TSS, in line with our observations in LβT2 cells. Three ESTs from embryonic day 14.5 Rathke’s pouch (the pituitary primordium), however, have predicted UTRs of 220 (acc. # CJ178295) or 373 bp (acc. # CJ178954 or CJ175512). These longer UTRs are more in line with what was previously reported in the human (418 bp; acc. # NG_012454) and caprine [311 bp, ref. [28]] *FOXL2* mRNAs. The source of these discrepancies in UTR length is unresolved at present, but might reflect differences between tissues, species, development stage, and methodologies. All of our RACE clones mapped the TSS to the same nucleotide; however, if the 373 bp UTR from the Rathke’s pouch ESTs is accurate, then our shortest reporter, -187/+7, would lack 5’ flanking sequence. In contrast, the minimal reporter, -432/+7, would contain ~220 bp of proximal promoter. It is possible that this could have contributed to the difference in activity between these two reporters. This would not, however, diminish the discovery of the CpG island in the 5’ UTR and proximal promoter of the *Foxl2* gene, and the potential role of its methylation status in the gene’s transcriptional activity in some cell types.

Though DNA methylation is generally considered a repressive mark, its mechanism of transcriptional inhibition is context-dependent. For example, methylation of a cytosine within a *cis*-regulatory element can impair transcription factor binding [e.g., [64,69]]. Alternatively, methyl-binding proteins, such as MeCP2, can recruit transcriptional repressors and chromatin remodeling proteins to inhibit transcription [e.g., [70-75]]. Though we show that DNA methylation silences *Foxl2* promoter-reporter activity in heterologous and homologous cells, we did not establish the underlying mechanism of transcriptional repression. That said, M.*Hha*I, which methylated the reporter at only 4 of the 40 CpGs within the -432/+7 interval, silenced transcriptional activity by ~70% and three of these CpGs are conserved at the equivalent positions in the human *FOXL2* gene. These observations might suggest the presence of important *cis*-regulatory sequences at these positions. We reasoned that if methyl-cytosine blocks transcription factor binding, substitution of another bp for cytosine should similarly disrupt protein binding. However, mutation of these 4 sites (C to A) alone or together did not diminish activity of the -432/+7 reporter (data not shown). In addition, the -432/+7 sequence in mouse and corresponding sequence in human are highly conserved; however, only 26 (of 40 in mouse and 39 in human) of the CpGs are common to both species. Thus, there appears to be a strong selective pressure to maintain a high density of CpGs within the proximal promoter and 5’ UTR of the *Foxl2/FOXL2* gene, but the absolute location of the CpGs might be less important. Collectively, therefore, we would predict that recruitment of transcriptional repressors rather than blockade of transcription factor binding more likely explains how DNA methylation might repress *Foxl2* transcription. Clearly, this will require experimental validation.

Though proximal promoter methylation can silence *Foxl2* transcription, both our own and other data indicate that this mechanism alone cannot fully explain cell-specific expression of the gene. First, whereas some heterologous cell lines exhibited relative *Foxl2* promoter hypermethylation (NIH3T3, AtT20, GT1-7), others (C2C12, TtT/GF) showed methylation patterns similar to homologous cell lines. In addition, though heterologous tissues such as heart, liver, and testis appeared to show greater *Foxl2* proximal promoter methylation than the homologous ovary, methylation was far from complete (20-35%). Whereas it is true that the extent of methylation in ovary (10%) might be misleading because the samples included both homologous (granulosa) and heterologous cells (e.g., theca and oocytes), one might have predicted more extensive methylation in the heterologous tissues if this alone accounted for cell-type-specific expression. In addition, the *Foxl2* promoter was hypomethylated in both homologous gonadotropes and heterologous pituitary cell types. Though the latter included thyrotropes, these represent less than 5% of the total pituitary cell population; therefore, it is unlikely that promoter hypomethylation in these cells diluted promoter hypermethylation in the other, more abundant pituitary cell types. Instead, our data appear to be consistent with earlier reports showing discrepancies between CpG methylation in tissues and corresponding immortalized cells lines (e.g., [76-79]). For example, the CpG-rich *CFTR* promoter is hypomethylated in tissues regardless of the level of *CFTR* gene expression, whereas there appears to be an inverse correlation between the gene’s promoter methylation and expression in cell lines [80]. Consequently, although our data indicate that CpG methylation can silence *Foxl2* promoter activity *in vitro*, other mechanisms beyond proximal promoter hypomethylation must contribute to cell-type specific *Foxl2* expression in gonadotropes and likely other homologous cell types *in vivo*.

Investigations in PIS goats and in BPES human patients clearly indicate roles for distal enhancers and repressors in *FOXL2* expression. As described in the Introduction, a *cis*-regulatory sequence nearly 300 kb upstream of the *FOXL2* coding sequence acts to repress expression in the horn buds and to activate transcription in the ovary, but does not play a role in the developing eyelid of goats [27]. Similarly, several cases of BPES are linked to microdeletions and/or translocations upstream or downstream of the *FOXL2* coding sequence [e.g., [13-21]]. Thus, these data converge to indicate that regulatory elements (repressors and enhancers), often at great distances from the *FOXL2* gene, regulate expression and likely do so in cell-specific fashion. The challenge for future investigations will be to identify the precise location and nature of distal regulatory sequences [e.g., using chromosomal conformation capture or transgenic approaches; e.g., [81-83]] to understand if or how they associate with the proximal promoter [e.g., [21]], and to characterize if or how promoter methylation affects these interactions in both homologous and heterologous cells.

## Supporting Information

Figure S1 **The murine *Foxl2* 5’ flaking sequence confers reporter activity in both orientations.** A) LβT2 or B) NIH3T3 cells were transfected with empty vector (pGL3-Basic) or -677/+7 of the murine Foxl2 5’ flanking sequence inserted into pGL3-Basic in the sense or antisense directions. Transcriptional activity was measured using luciferase assays. The data reflect the means of 2 or 3 independent experiments and are presented with empty vector activity set to 1.
(PDF)

Figure S2 ***In vitro* methylation silences *Foxl2* promoter reporter activity in LβT2 and NIH3T3 cells.** The pCpGL-CMV/EF1, empty pCpGL-Basic, or pCpGL-432/+7 murine *Foxl2* vectors were treated in vitro with *M*.*Sss*I. Mock methylated plasmids were exposed to the identical treatment but without enzyme. Plasmids were transfected in triplicate into A) LβT2 (n=4) or B) NIH3T3 (n=3) cells. The data reflect the means of independent experiments (+SEM). For purposes of comparison, the mock methylated plasmid was set to 1 for each reporter.
(PDF)

Figure S3 **Decitabine treatment fails to induce *Foxl2* expression in NIH3T3 cells.** A) RT-PCR analysis of *Foxl2* (top), *Hdc* (middle), and *Rpl19* (bottom) expression in RNA from NIH3T3 cells treated with vehicle (-) or 10 µM decitabine (+) for 8 days. Data are from a representative of a total of 6 replicate experiments. RNA from untreated LβT2 cells was included as a positive control for the *Foxl2* primer set. *Rpl19* was used as a control for RNA integrity and RT efficiency in all samples. Percent methylation of the indicated segments of the (B) *Hdc* and (C) *Foxl2* promoters was assessed by qAMP. Data are from a representative of two replicate experiments, which yielded comparable results.
(PDF)

Figure S4 **FOXL2 is expressed in gonadotrope-like, but not other cell lines.** A) RT-PCR analysis of *Foxl2* mRNA expression in the indicated cell lines. *Rpl19* was used as a loading control. Murine *Foxl2* expression plasmid was used as a positive control for the *Foxl2* primer set. B) Immunoblot (IB) analysis of FOXL2 protein expression in the indicated cell lines. β-actin (ACTB) was used as a loading control.
(PDF)
