# Supplementary figures and images for: The CpG Island in the Murine Foxl2 Proximal Promoter Is Differentially Methylated in Primary and Immortalized Cells
Source: PLoS One. 2013 Oct 2;8(10):e76642. doi: 10.1371/journal.pone.0076642 (PMC3788739; doi:10.1371/journal.pone.0076642)

Figure S1

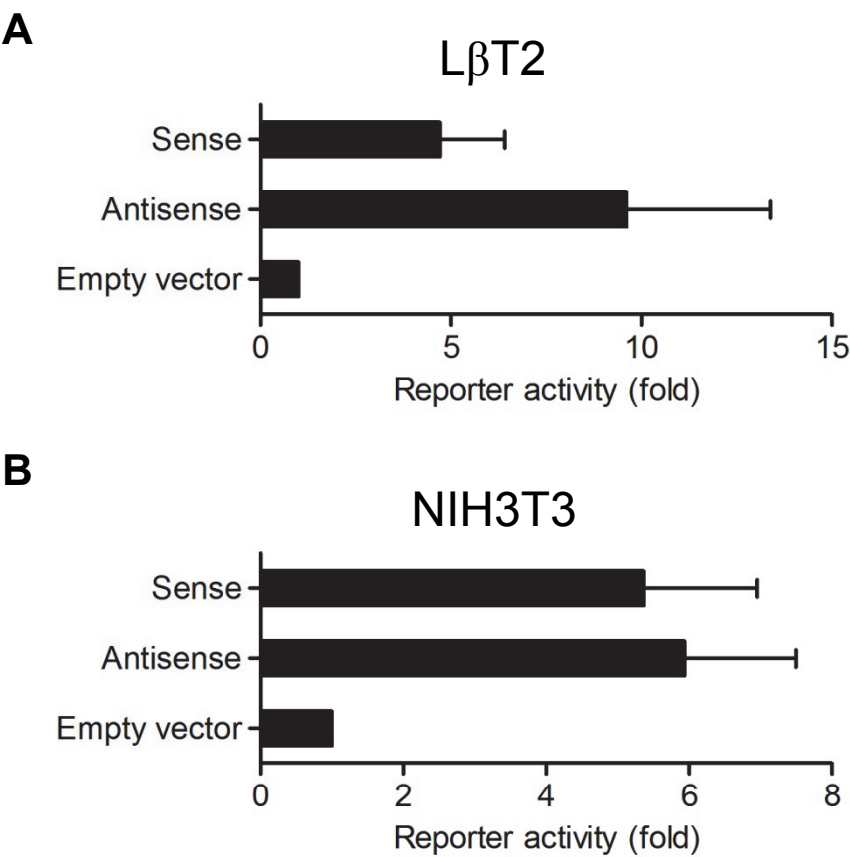

Supplement: Figure S1 — The murine Foxl2 5’ flaking sequence confers reporter activity in both orientations. A) LβT2 or B) NIH3T3 cells were transfected with empty vector (pGL3-Basic) or -677/+7 of the murine Foxl2 5’ flanking sequence inserted into pGL3-Basic in the sense or antisense directions. Transcriptional activity was measured using luciferase assays. The data reflect the means of 2 or 3 independent experiments and are presented with empty vector activity set to 1. (PDF) [file pone.0076642.s001.pdf]

Figure S2

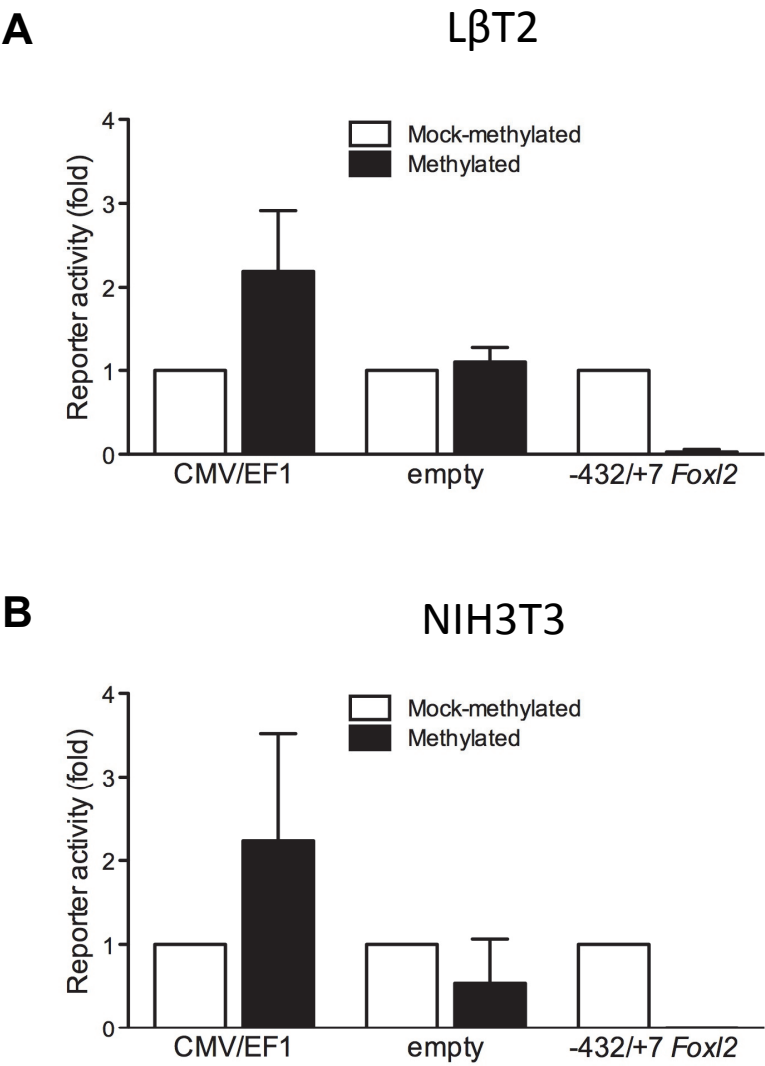

Supplement: Figure S2 — In vitro methylation silences Foxl2 promoter reporter activity in LβT2 and NIH3T3 cells. The pCpGL-CMV/EF1, empty pCpGL-Basic, or pCpGL-432/+7 murine Foxl2 vectors were treated in vitro with M.SssI. Mock methylated plasmids were exposed to the identical treatment but without enzyme. Plasmids were transfected in triplicate into A) LβT2 (n=4) or B) NIH3T3 (n=3) cells. The data reflect the means of independent experiments (+SEM). For purposes of comparison, the mock methylated plasmid was set to 1 for each reporter. (PDF) [file pone.0076642.s002.pdf]

Figure S3

A

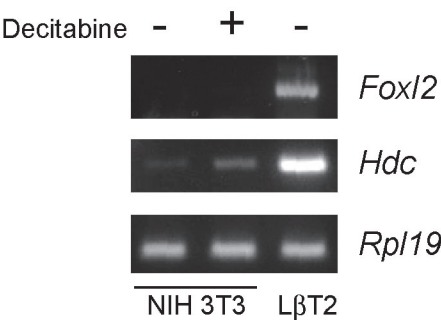

B

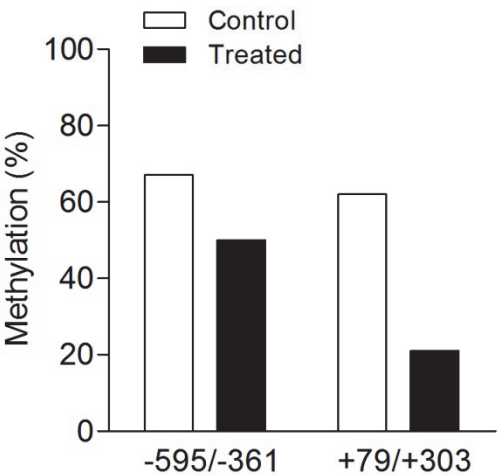

C

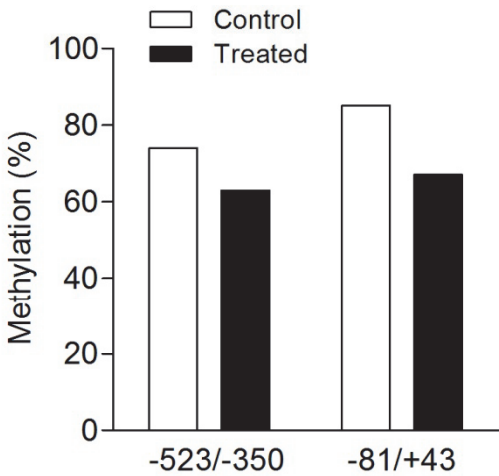

Supplement: Figure S3 — Decitabine treatment fails to induce Foxl2 expression in NIH3T3 cells. A) RT-PCR analysis of Foxl2 (top), Hdc (middle), and Rpl19 (bottom) expression in RNA from NIH3T3 cells treated with vehicle (-) or 10 µM decitabine (+) for 8 days. Data are from a representative of a total of 6 replicate experiments. RNA from untreated LβT2 cells was included as a positive control for the Foxl2 primer set. Rpl19 was used as a control for RNA integrity and RT efficiency in all samples. Percent methylation of the indicated segments of the (B) Hdc and (C) Foxl2 promoters was assessed by qAMP. Data are from a representative of two replicate experiments, which yielded comparable results. (PDF) [file pone.0076642.s003.pdf]

Figure S4

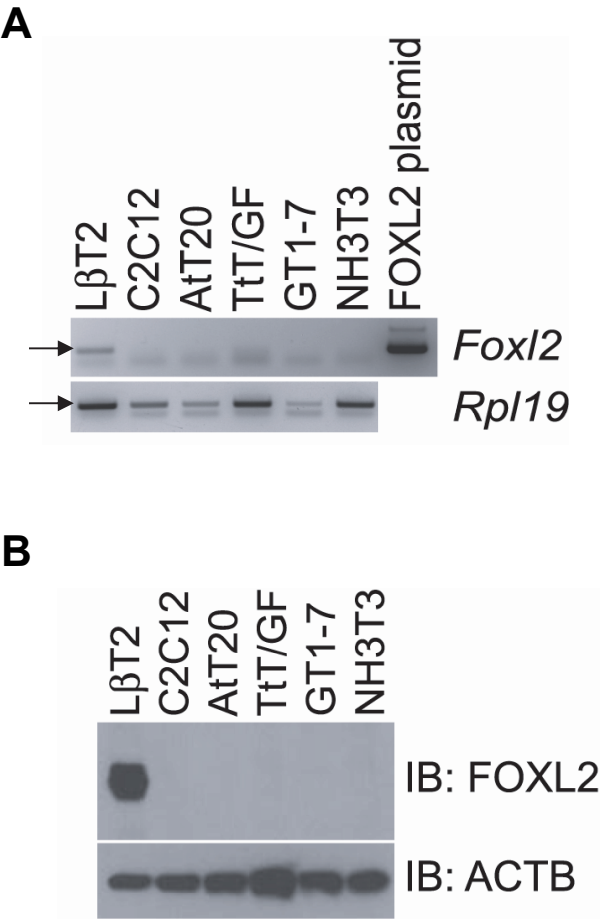

Supplement: Figure S4 — FOXL2 is expressed in gonadotrope-like, but not other cell lines. A) RT-PCR analysis of Foxl2 mRNA expression in the indicated cell lines. Rpl19 was used as a loading control. Murine Foxl2 expression plasmid was used as a positive control for the Foxl2 primer set. B) Immunoblot (IB) analysis of FOXL2 protein expression in the indicated cell lines. β-actin (ACTB) was used as a loading control. (PDF) [file pone.0076642.s004.pdf]
